# 

**Published:** 1906-05-19

**Authors:** 


CONTENTS.

**Special Societies and the Amalgamation Scheme** 115
**Physical Deterioration** - - - - - - - - 116
**Annotations** - - - - - - - - - - - 117
**John Banister: the Troubles of a Seventeenth Century Oculist** - - - - - - - - - 118
**Hospital Clinics—**
The Prognosis in a Case of Cerebral Hæmorrhage - - - 119
Pharmacopœial Doses - - - - - - - - - 120
Ataxia in Childhood - - - - - - - - - 121
Hæmorrhagic Pancreatitis - - - - - - - - 122
**Progress in Medicine and Surgery—**
Carcinoma - - - - - - - - - - - 123
Diseases of the Digestive Organs - - - - - - - 124
**The Book World of Medicine and Science** - - 125
**New Appliances and Things Medical** - - - - 125
**Medical Appointments and Vacancies** - - - 125
**Hospital Administration—**
The Hospital Movement in the United States - - - 126
Current Hospital Topics - - - - - - - - 128
North-Eastern Hospital for Children - - - - - 128
Portugal as a Holiday and Health Resort - - - - - 130

*NURSING SECTION.*

**Notes on News from the Nursing World—**
Miss Nightingale's Birthday—English Nurses in Belgium—Censure of a Nurse by a Jury—The Provocations of Mental Nurses—Fourteen Yorkshire Workhouses without a Night Nurse—Imperial Military Nursing Service—The Nurses of St. George's Hospital—The Royal National Pension Fund—Thirty-eight Years' Service—Princess Christian's Trained Nurses—Stallholders at the Elizabethan Fair—British Nurses' Association—Birmingham General Hospital League—Home for Nurses at Keswick—A Friend of Nurses—Clapham Maternity Hospital—The Nurses of Camberwell Poor-law Infirmary—Workmen and Queen's Nurses—A Nursing Institute for Tiverton—Short Items - - - - - - - - 99-102
**The Nursing Outlook** - - - - - - - - 103
**The Care and Nursing of the Insane** - - - - 104
**The Nurses' Clinic** - - - - - - - - - 105
**The Best Method of Keeping Helpless Patients Dry** - - - - - - - - - - - 106
**Incidents in a Nurse's Life** - - - - - - - 107
**Sir William Bennett on Nursing Associations** - 107
**East-End Mothers' Lying-in Home** - - - - 108
**Presentations** - - - - - - - - - - - 108
**Everybody's Opinion** - - - - - - - - 108
**Appointments** - - - - - - - - - - - 109
**Death in Our Ranks** - - - - - - - - - 109
**A Book and Its Story** - - - - - - - - 110
**Travel Notes and Queries** - - - - - - - 111
**Notes and Queries** - - - - - - - - - - 111
**For Reading to the Sick** - - - - - - - - 112

